# Validation of the Brief Children’s Depression Rating Scale in Children Treated With Selegiline Transdermal Patch vs Placebo

**DOI:** 10.1016/j.jaacop.2025.11.010

**Published:** 2025-12-01

**Authors:** Milica Barac, Paul A. Nakonezny, Paul E. Croarkin, Arjun P. Athreya, Sophia M. Jones, Sarah M. Hergert, Graham J. Emslie

**Affiliations:** a Mayo Clinic, Rochester, Minnesota; b Peter O’Donnell Jr. School of Public Health, University of Texas Southwestern Medical Center, Dallas, Texas; c Peter O’Donnell Jr. Brain Institute, University of Texas Southwestern Medical Center, Dallas, Texas

**Keywords:** adolescents, children, major depressive disorder, measurement-based care, psychometric rating scale, selegiline

## Abstract

**Objective:**

Brief adaptations of existing rating scales may have advantages for research and clinical practice. This study examined the relative performance and psychometric properties of the 5-item Brief Children’s Depression Rating Scale–Revised (BCDRS-R5) in the context of a placebo-controlled clinical trial.

**Method:**

The present study evaluated the internal consistency, scale dimensionality, relative performance in identifying remission and response, and sensitivity to change of the BCDRS-R5 in outpatients (N = 296) 12 to 17 years of age with major depressive disorder enrolled in a randomized, double-blind, placebo-controlled parallel group trial of the selegiline transdermal patch.

**Results:**

The Cronbach coefficient alpha of the BCDRS-R5 ranged from 0.679 at baseline to 0.866 at week 12. A 1-factor solution for the BCDRS-R5 is suggested by the principal component analysis. The BCDRS-R5 demonstrated good accuracy in detecting response and remission.

**Conclusion:**

The BCDRS-R5 has good performance and is sensitive to change in symptom severity over 12 weeks of treatment, with either medication or placebo, when compared with the longer, standard CDRS-R17.

**Clinical trial registration information:**

Phase IV:Safety and Efficacy of EMSAM in Adolescents With Major Depression; https://clinicaltrials.gov/study/NCT00531947

**Diversity & Inclusion Statement:**

We actively worked to promote sex and gender balance in our author group. We actively worked to promote inclusion of historically underrepresented racial and/or ethnic groups in science in our author group. While citing references scientifically relevant for this work, we also actively worked to promote sex and gender balance in our reference list. While citing references scientifically relevant for this work, we also actively worked to promote inclusion of historically underrepresented racial and/or ethnic groups in science in our reference list.

## Plain language summary

Brief adaptations of clinical rating scales may help streamline research and measurement-based care. This study examined data from a prior clinical trial of selegiline for adolescents with depression (N = 296). The goal was to study the performance of a brief, 5-item version of the Children’s Depression Rating Scale–Revised (BCDRS-R5). This rating scale appeared to have similar properties as compared to the standard 17-item Children’s Depression Rating Scale–Revised (CDRS-R17) from which the BCDRS-R5 was derived. This study is the first psychometric validation of the BCDRS-R5 in the context of a placebo randomized controlled trial for adolescents with major depressive disorder. Results suggest the brief scale has good performance and is sensitive to change in symptom severity over time.

Successful implementation of measurement-based care and optimal clinical research efforts focused on major depressive disorder (MDD) will require streamlined, reliable, and valid standardized rating scales for assessing depressive symptom severity in children. Promising approaches to address this need have led to brief versions of standard rating scales to mitigate time burden and to improve accessibility, reliability, and validity.^1^, ^2^, ^3^ Ideal rating scales would seamlessly meet the needs of families, clinicians, and researchers.^4^, ^5^, ^6^, ^7^ Brief adaptations of clinical rating scales would also support the ongoing transformation of clinical practice through decentralized care models and digital platforms.^8^^,^^9^

The Patient Health Questionnaire–9 (PHQ-9)^10^ and its adaptations such as the Patient Health Questionnaire–9 Modified (PHQ-9M)^11^ are reliable^12^ and widely used screening tools. The Quick Inventory of Depressive Symptoms (QIDS),^13^ and the Kutcher Adolescent Depression Scale (KADS)^14^ are other self-reported options for assessments of depressive symptom severity. The CDRS-R17, which is a clinician-administered scale, is frequently used as an outcome measure for clinical trials focused on youth with depression.^15^^,^^16^ Common drawbacks of such rating scales include the time burden associated with administration,^11^ and the precision of detecting treatment response.^17^ An assessment tool for depressive symptom severity that meets needs for acceptability, efficiency, reliability, and validity^18^ while mitigating time burden would be ideal.

Previous research of clinician-administered scales has examined the brief version of the QIDS for assessing MDD symptom severity among adults.^19^ Other work has demonstrated that the 6-item version of the Hamilton Rating Scale for Depression (HRSD), when compared to the standard 17-item HRSD, accounted for most of the variance in depressive symptoms.^20^ The 2-item version of the PHQ self-report measure demonstrated good sensitivity and specificity for detecting major depression in youth.^21^ Prior work has examined the 5-item Brief Children’s Depression Rating Scale–Revised (BCDRS-R5) in adolescents with MDD^22^; however, the current study is the first to replicate and examine the performance of the brief BCDRS-R5 in the context of a placebo-controlled trial so as to further strengthen the internal and external validity of the BCDRS-R5.^22^^,^^23^

To further advance efforts on validating the brief BCDRS-R5, this study examined its use in a secondary analysis of adolescents with MDD receiving treatment with the selegiline transdermal patch (EMSAM, Somerset Pharmaceuticals, Inc) or placebo. The psychometric properties and performance of the BCDRS-R5 were examined. In addition, a clinically relevant depressive symptom severity threshold for remission was defined. It was hypothesized that the BCDRS-R5 would offer comparable psychometric properties and performance to the longer, standard version of the CDRS-R17 interview.

## Method

This study used data obtained from a randomized, double-blind, placebo-controlled, flexible-dose, parallel-group trial that was conducted at 26 clinical investigational sites in the United States to examine the efficacy and safety of the selegiline transdermal patch in adolescents with moderate or severe MDD. In brief, the current study included a subsample of 296 outpatient youths, 12 to 17 years of age, who met criteria for moderate to severe MDD for at least 4 weeks but less than 2 years (based upon the Kiddie Schedule for Affective Disorders and Schizophrenia for School Aged Children (K-SADS) and a CDRS-R17 total score of ≥45). Clinical ratings with the CDRS-R17 were completed during weeks 1, 2, 4, 5, 6, 8, 9, 10, and 12. Of the original 152 and 156 adolescents from the selegiline RCT who were randomized to selegiline transdermal patch or placebo, respectively, 7 patients from the selegiline group and 5 patients from the placebo group (for a total of 12 patients) were excluded here because they failed to return following the baseline visit; thus, this left us with 296 total adolescents for this study (post hoc secondary analysis). A detailed description of the full methodology and outcomes has been previously reported.^24^^,^^25^

### Measures

The schedule of assessments, and overall study findings have been reported elsewhere.^24^ For the current study, however, we used outcomes of symptom severity collected at baseline and at weeks 4, 6, and 12, and symptom severity was assessed using the CDRS-R17.^16^^,^^26^ The CDRS-R17 is a 17-item, semi-structured interview administered by a trained professional. The recommended mode is to obtain a multi-informant report by interviewing the child and their parent (or another adult who knows the child well enough to provide reliable information). It typically takes between 20 and 30 minutes to complete a CDRS-R17 interview. The first 14 items of the CDRS-R17 are rated from an interview with the patient and parent, and the last 3 items are scored from rater observations (facial affect, speech, and hypoactivity). The CDRS-R17 items are rated on 5-point (items 4, 5, and 16) or 7-point (items 1-3, 6-15, and 17) Likert-type scales. The clinician provides an overall summary score based on interviews and scores of the parent and child. Total scores can range from 17 to 113, with a greater score representing greater depressive symptom severity.

This study also used the BCDRS-R5 (total score range, 5-35).^22^^,^^23^ The BCDRS-R5 was derived from the CDRS-R17 and included identical items 2 (difficulty having fun), 3 (social withdrawal), 10 (low self-esteem), 11 (depressed feelings), and 15 (depressed facial affect) from the CDRS-R17.^22^ Thus, we simply used the ratings from the CDRS-R17 and the corresponding ratings from the 5 items that make up the BCDRS-R5 from the same interviewer and same subject at each assessment visit. Details and psychometric properties on this brief scale can be found elsewhere.^22^^,^^23^ An item response theory (IRT) analysis, which was implemented using the graded response model, confirmed the 5-items for BCDRS-R5 used in this study (Table S1, available online).

Remission status was defined at each visit (weeks 4, 6, and 12) as a total score of ≤28, based on the gold standard CDRS-R17.^1^^,^^27^^,^^28^ The remission category or threshold for the BCDRS-R5 that emerged from the ROC analysis was compared against this gold standard definition of remission. Treatment response, irrespective of remission, was operationally defined as a decrease of at least 50% in total score (symptom severity) on the CDRS-R17 and the BCDRS-R5 from baseline to weeks 4, 6, and 12, respectively.

### Multiple Imputation for Missing Values

Missing values observed for the CDRS-R17 at weeks 4, 6, and 12 occurred in no more than about 10%, 17%, and 25% of the sample, respectively, and were imputed. Missing values (with an assumed arbitrary missing pattern) were imputed via 500 burn-in iterations (samples) using Fully Conditional Specification along with the discriminant method of the PROC MI procedures in SAS software, version 9.4. The Little χ^2^ test^29^ supported the MCAR assumption (χ^2^ = 151.08, *p* = .369).

### Statistical Analysis

Classical test theory (CTT) analysis was used to determine the mean-item total correlations, the scale mean, and standard deviation, as well as the internal consistency (Cronbach coefficient α) for each scale. Principal component analysis (PCA), with varimax rotation, evaluated the dimensionality of the BCDRS-R5. The CTT analysis was implemented at baseline and at weeks 6 and 12.

The optimal cut point for the BCDRS-R5 (based on the Youden index) in discriminating remission status at the completion of 6 weeks of treatment, based on the gold standard definition of remission of CDRS-R17 total ≤28,^30^^,^^31^ was determined by a receiver operating characteristic (ROC) analysis. Sensitivity and specificity as well as the area under the curve (AUC) were reported for the optimal cut point. We selected a 6-week time point, as this is consistent with the clinical timeline for evaluating remission during the acute treatment of youth with MDD. Moreover, the 6-week mark allows us to compare the cut point to that found in our previous study on the BCDRS-R5.^23^

The frequency distribution of remission and response status from baseline to weeks 4, 6, and 12 were reported. Categories of benefit included treatment response irrespective of remission (≥50% reduction in symptom severity from baseline) as well as remission status that emerged from the ROC analysis. The strength of agreement between BCDRS-R5 and CDRS-R17 was assessed by sensitivity, specificity, positive predictive value (PPV), negative predictive value (NPV), classification accuracy rate, and misclassification (error) rate for remission and response status.

Finally, the sensitivity of the CDRS-R17 and the BCDRS-R5 to change in symptom severity over 12 weeks of treatment with the selegiline transdermal patch vs placebo was evaluated by computing the percent change from baseline to weeks 4, 6, and 12. The dependent-samples *t* test was used to test for differences in the mean percent change at each timepoint. The Cohen *d* (which accounted for the within-subjects correlation of the paired values) was also calculated and interpreted as the effect size estimator for the relative magnitude of change.

Statistical analyses were performed using SAS software, version 9.4 (SAS Institute, Inc, Cary, NC) and MedCalc for Windows, version 23.0.9 (MedCalc Software, Ostend, Belgium). The procedures in MedCalc were used to conduct the ROC analyses. The level of significance for all tests was set at α = 0.05 (2-tailed).

## Results

### Participant Characteristics

The sample in this study consisted of 296 participants, which included 64.9% female adolescents, with a mean age of 14.7 ± 1.6 years (range, 12-17 years). Mean CDRS-R17 total at baseline was 60.1 ± 11.5, which reflected moderate symptom severity. About 48% of the youth who received the selegiline transdermal patch attained remission (CDRS-R17 total score ≤28) following 12 weeks of treatment. Participant characteristics are reported in Table 1.

**Table 1:** Demographic and Clinical Characteristics of the Study Sample

Participant characteristic	Study sample	Selegiline	Placebo
	(N = 296)	(n = 145)	(n = 151)
**Demographics**			
Age, y, mean ±SD	14.7 ± 1.6	14.8 ± 1.6	14.7 ± 1.6
Sex, % (n)			
Male	35.1 (104)	38.6 (56)	31.8 (48)
Female	64.9 (192)	61.4 (89)	68.2 (103)
Race,^a^ % (n)			
Asian	1.0 (3)	0.7 (1)	1.3 (2)
Black	25.7 (76)	31.0 (45)	20.6 (31)
Caucasian/White	47.6 (141)	44.8 (65)	50.3 (76)
Hispanic	22.3 (66)	20.7 (30)	23.8 (36)
Mixed race	2.7 (8)	2.1 (3)	3.3 (5)
Native American	0.7 (2)	0.7 (1)	0.7 (1)
Clinical characteristics			
History of recurrent depression, % (n)	40.5 (120)	41.4 (60)	39.7 (60)
Response at week 12,^b^ % (n)	72.3 (296)	73.8 (145)	70.9 (151)
CDRS-R_17_ total at baseline, mean ± SD	60.1 ± 11.5	59.3 ± 10.7	60.6 ± 12.1
CDRS-R_17_ total at week 4, mean ± SD	44.9 ± 14.2	44.3 ± 13.5	45.5 ± 14.8
CDRS-R_17_ total at week 6, mean ± SD	37.6 ± 13.5	37.5 ± 12.4	37.7 ± 14.5
CDRS-R_17_ total at week 12, mean ± SD	32.4 ± 13.1	31.7 ± 12.1	33.1 ± 13.9
BCDRS-R_5_ at baseline, mean ± SD	21.5 ± 4.3	21.4 ± 4.3	21.5 ± 4.4
BCDRS-R_5_ at week 4, mean ± SD	15.8 ± 5.7	15.7 ± 5.5	15.9 ± 5.8
BCDRS-R_5_ at week 6, mean ± SD	13.2 ± 5.6	13.4 ± 5.4	13.0 ± 5.9
BCDRS-R_5_ at week 12, mean ± SD	10.4 ± 5.1	10.5 ± 5.2	10.2 ± 5.1

Note: The means presented in this table are the sample means. BCDRS-R = Brief Children’s Depression Rating Scale–Revised; CDRS-R = Children’s Depression Rating Scale–Revised; selegiline = selegiline transdermal patch (EMSAM).

a This was the racial categorization in the original publication and dataset.

b Response was operationally defined as a ≥50% reduction in symptom severity (CDRS-R17 total) from baseline to the end of the 12-week study/exit.

### Internal Consistency and Scale Dimensionality

The Cronbach coefficient α (internal consistency) of the BCDRS-R5 was 0.679 at baseline and 0.866 at week 12. Cronbach coefficient α values for CDRS-R17 at baseline and week 12 were 0.797 and 0.902, respectively. The PCA suggested a 1-factor solution for the BCDRS-R5. Total variance explained by the sole principal component ranged from 44.68% at baseline to 65.61% at week 12. The CTT results are reported in Table S1, available online.

### ROC Analysis

The ROC analysis was used to determine the optimal cut point for the BCDRS-R5 in discriminating remission status at the completion of 6 weeks of treatment based on the gold standard CDRS-R17 total ≤28.^30^^,^^31^ The ROC analysis found that the remission categories (optimal cut points) that emerged were defined as a score of ≤10 on the BCDRS-R5 in the overall sample (AUC = 0.975, sensitivity = 97.30%, specificity = 88.37%), a score of ≤10 on the BCDRS-R5 for those who received the selegiline transdermal patch (AUC = 0.983, sensitivity = 96.87%, specificity = 94.44%), and a score of ≤10 on the BCDRS-R5 for those who received placebo (AUC = 0.967, sensitivity = 95.24%, specificity = 86.59%).

### Comparisons for Categories of Benefit

**Figure 1:**
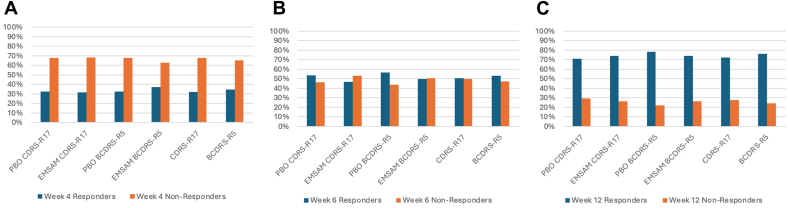
Percentage of Participants Who Were Responders vs Nonresponders From Baseline to Weeks 4 (A), 6 (B), and 12 (C) ***Note:****The Response category was defined as a ≥50% reduction in symptom severity from baseline to weeks 4 (A), 6 (B), and 12 (C).*

**Figure 2:**
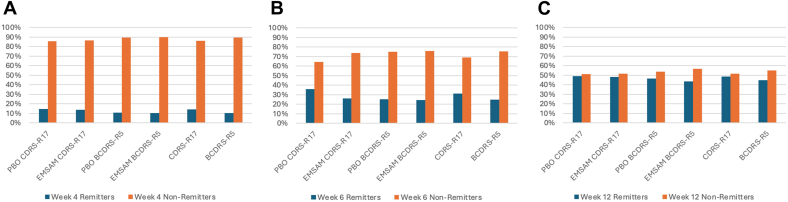
Percentage of Participants Who Were Remitters vs Nonremitters From Baseline to Weeks 4 (A), 6 (B), and 12 (C) ***Note:****The Remission category was defined as a score of ≤10 on the BCDRS-R and ≤28 on the CDRS-R17. BCDRS-R = Brief Children’s Depression Rating Scale–Revised; CDRS-R = Children’s Depression Rating Scale–Revised.*

Categories of benefit included treatment response irrespective of remission and remission status that emerged from the ROC analysis. Figures 1 and 2 show the frequency distribution of response and remission status from baseline through week 12. As shown in Table 2, from baseline to weeks 4, 6, and 12, respectively, the BCDRS-R5 vs CDRS-R17 demonstrated good accuracy in detecting remission status (correct classification rate ranged from 79.39% to 93.58%) and response status (correct classification rate ranged from 80.74% to 85.13%).

**Table 2:** Strength of Agreement for Remission and Response (Baseline to Week 12)

Index of Agreement	(n = 296)	(n = 296)	(n = 296)	(n = 296)	(n = 296)	(n = 296)
	Baseline to Week 4	Baseline to Week 4	Baseline to Week 6	Baseline to Week 6	Baseline to Week 12	Baseline to Week 12
	CDRS-R17	CDRS-R17	CDRS-R17	CDRS-R17	CDRS-R17	CDRS-R17
	BCDRS-R5	BCDRS-R5	BCDRS-R5	BCDRS-R5	BCDRS-R5	BCDRS-R5
	Remission %	Response %	Remission %	Response %	Remission %	Response %
Positive predictive value (PPV)	87.10	74.76	84.93	81.53	81.20	84.89
Negative predictive value (NPV)	94.34	90.67	86.55	84.89	77.91	67.61
Sensitivity	64.29	81.05	67.39	85.91	75.00	89.25
Specificity	98.43	87.06	94.61	80.27	83.55	58.54
Accuracy or correct classification rate	93.58	85.13	86.14	83.11	79.39	80.74
Error rate or misclassification rate	6.42	14.87	13.86	16.89	20.61	19.26

Note: The Remission category was defined as a score of ≤10 on the BCDRS-R5 and ≤28 on the CDRS-R17 at weeks 4, 6, and 12. The Response category was defined as a ≥50% reduction in symptom severity from baseline to weeks 4, 6, and 12. BCDRS-R = Brief Children’s Depression Rating Scale–Revised; CDRS-R = Children’s Depression Rating Scale–Revised.

### Sensitivity to Change Over 12 Weeks of Treatment

The sensitivity of the BCDRS-R5 and the CDRS-R17 to change in symptom severity over 12 weeks of treatment, with the selegiline transdermal patch or placebo, was evaluated by the percent change from baseline to weeks 4, 6, and 12. These results, as shown in Table 3 for the selegiline transdermal patch and Table 4 for placebo, revealed that the relative magnitude of sensitivity of each of the two scales to change in depressive symptom severity (as evaluated by the Cohen d) was very similar.

**Table 3:** Mean Percent Change Over 12 Weeks of Selegiline Transdermal Patch (EMSAM) Treatment for the Two Scales

Clinical outcome (n = 145)	Baseline to week 4			Baseline to week 6			Baseline to week 12		
	Mean % change (SD)	95% CI	*d*	Mean % change (SD)	95% CI	*D*	Mean % change (SD)	95% CI	*d*
CDRS-R17	–35.07 (28.79)	–39.80 to –30.35	1.29	–48.69 (33.69)	–54.22 to –43.16	1.92	–64.03 (28.79)	–68.75 to –59.30	2.93
BCDRS-R5	–34.18 (32.33)	–39.48 to –28.87	1.13	–47.31 (34.40)	–52.96 to –41.67	1.78	–64.56 (33.11)	–70.01 to –59.12	2.64

Note: Dependent-samples t test was used to test for differences in the mean percent change at each time period. All *p* values were <.0001. *d* = Cohen *d* effect size estimator (which accounted for the within-subjects correlation of the paired values).

**Table 4:** Mean Percent Change Over 12 Weeks of Placebo Treatment for the Two Scales

Clinical outcome (n = 151)	Baseline to week 4			Baseline to week 6			Baseline to week 12		
	Mean % change (SD)	95% CI	*d*	Mean % change (SD)	95% CI	*D*	Mean % change (SD)	95% CI	*d*
CDRS-R17	–34.21 (29.74)	–38.99 to –29.42	1.12	–51.64 (31.11)	–56.74 to –46.64	1.86	–62.61 (30.02)	–67.44 to –57.78	2.55
BCDRS-R5	–34.92 (31.96)	–40.06 to –29.78	1.10	–51.35 (33.48)	–56.74 to –45.97	1.76	–68.37 (29.62)	–73.13 to –63.61	2.95

Note: Dependent-samples t test was used to test for differences in the mean percent change at each time period. All *p* values were <.0001. *d* = Cohen *d* effect size estimator (which accounted for the within-subjects correlation of the paired values).

## Discussion

This study investigated the performance and psychometric properties of the BCDRS-R5 in the context of a placebo-controlled trial for adolescents with MDD. Along with internal consistency, the BCDRS-R5 demonstrated sensitivity to change in symptom severity over 12 weeks of treatment with the selegiline transdermal patch or placebo. A 1-factor solution for the BCDRS-R5 was supported following the principal component analysis. The remission category defined as a score ≤10 on the BCDRS-R5 is consistent with prior work^23^ that examined various scales, including the BCDRS-R5, in adolescents with major depressive disorder.^13^^,^^32^ This study demonstrated that the BCDRS-R5 is accurate in detecting remission and response compared to the CDRS-R17. This is consistent with what was demonstrated in previous work.^23^

The present study contributes to the limited literature validating the BCDRS-R5 in adolescents with MDD. Previous work^22^ found that 5 CDRS-R17 items reflected disease severity for the adaptation of the BCDRS-R5. Furthermore, a prior machine learning–based effort showed that 4- to 6-week changes in severity of 4 of the 5 BCDRS-R5 items were also predictive of remission/response at 10 to 12 weeks.^33^ The current study as well as our prior work confirmed these 5 items via IRT analysis, and demonstrated that the BCDRS-R5 has favorable psychometric performance and is sensitive to change over 6 weeks in children and adolescents with MDD.^23^ The present study is the first psychometric validation of the BCDRS-R5 in the context of a randomized placebo-controlled trial for adolescents with MDD.

Previous research on the CDRS-R17 has proposed solutions with 2 to 5 factors,^2^ whereas this work, along with our prior work,^23^ support a unifactorial solution for the BCDRS-R5. A unifactorial rating scale for children with depression, sensitive to change in symptoms, could effectively differentiate clinical effects of an antidepressant and placebo in clinical trials.^34^, ^35^, ^36^, ^37^, ^38^, ^39^ The BCDRS-R5 could offer a unique benefit as a clinical rating scale, as it provides a reliable and valid framework while mitigating the time burden that is typical of standard rating scales. For example, the CDRS-R17 takes approximately 30 minutes to complete, whereas the BCDRS-R5 could be completed in about 10 minutes.^4^^,^^40^, ^41^, ^42^ Notably, the selegiline transdermal patch and placebo groups were very similar in terms of performance of the BCDRS-R5. The ideal applications of use for the BCDRS-R5 include concise clinic appointments, visits with digital therapeutic interventions, and remote visits. Implementing the BCDRS-R5 can help integrate parent and child reports and assess changes in symptom severity changes while undergoing treatment. An abbreviated assessment tool provides a crosswalk between scores on the longer and shorter versions, bridging the knowledge gap between research and clinical practice.

A possible limitation of this work is the use of a single sample, although it was across 26 sites. In addition, although the CDRS-R17 is widely used in clinical trials, no scale has perfect sensitivity and specificity for assessing symptom severity, and there is more to learn regarding its utility.^2^^,^^43^ Unfortunately, the present analyses relied on the CDRS-R17, and there were no other measures of depressive symptom severity to compare. This limited opportunities for external validation. The original clinical trial had an inclusion criterion of CDRS-R17 ≥45 to ensure that participants had moderate to severe depression, to mitigate placebo response, and to improve signal detection. For our study, this threshold for depressive symptom severity restricts the range of depressive symptom severity in our sample, may inflate reliability, and affects validity for a broader population. Future studies will need to examine more diverse samples, particularly youth at risk with subthreshold depressive symptoms. The BCDRS-R5 might not be useful in characterizing the heterogeneity of symptoms presented in depression. It should be noted that there are no items on the BCDRS that assess suicidal ideation or intent. Shorter scales such as the 5-item BCDRS-R5 may be more sensitive to this variability, which can result in lower alpha values at early time points. Furthermore, the internal consistency and percent variance of the BCDRS-R5 improved at week 12 (relative to baseline). Indeed, by weeks 6 and 12, as symptom patterns stabilized and clinical impressions became more consistent, both scales, CDRS-R17 and BCDRS-R5, demonstrated improved internal consistency. For the 5-item scale, by weeks 6 and 12, stronger inter-item correlations increased the Cronbach alpha to a level comparable to that of the full 17-item scale. This suggests that the BCDRS-R5 may be better at capturing subthreshold vs threshold MDD given the limited number of items. Future studies with more diverse and larger populations should be conducted to assist in further validating these instruments.^8^^,^^9^ The sample used in this work, however, was relatively diverse, with about 48% White and 52% non-White patients. In addition, 35.1% of the study sample was male, not far from the percentage of global male incidence cases of MDD (37.7%),^44^ further strengthening the generalizability of current findings.

In conclusion, the current study bolsters prior work^23^ suggesting that the BCDRS-R5, a novel, brief assessment tool, can potentially address unmet needs and mitigate time burden in contemporary clinical practice and research settings that are focused on the treatment of children and adolescents with depression. There is a need for a brief clinical and research instrument that is valid, reliable, and sensitive to accurately assess depressive symptom severity in children and adolescents. This study builds on our prior work^23^ and provides further evidence that the BCDRS-R5 has promise to effectively meet this need.

The next critical step in this line of research is to administer and evaluate the BCDRS-R5 independently from the full CDRS-R17. Doing so will allow us to further validate the scale in youth with MDD and to strengthen both its internal and external validity. Ultimately, our goal is to validate the BCDRS-R5 as a brief, efficient measure of depressive symptoms in youth—reducing burden for clinicians and patients while facilitating symptom monitoring across clinical and research contexts.

## CRediT authorship contribution statement

**Milica Barac:** Writing – review & editing, Writing – original draft, Visualization, Conceptualization. **Paul A. Nakonezny:** Writing – review & editing, Writing – original draft, Visualization, Supervision, Project administration, Methodology, Investigation, Formal analysis, Conceptualization. **Paul E. Croarkin:** Writing – review & editing, Writing – original draft, Supervision, Project administration, Methodology, Investigation, Conceptualization. **Arjun P. Athreya:** Writing – review & editing, Visualization, Conceptualization. **Sophia M. Jones:** Writing – review & editing, Visualization, Conceptualization. **Sarah M. Hergert:** Writing – review & editing, Visualization, Conceptualization. **Graham J. Emslie:** Writing – review & editing, Writing – original draft, Visualization, Investigation, Funding acquisition, Conceptualization.
