# Supplementary material for: Validation of the Brief Children’s Depression Rating Scale in Children Treated With Selegiline Transdermal Patch vs Placebo
Source: JAACAP Open. 2025 Dec 1;4(2):354–62. doi: 10.1016/j.jaacop.2025.11.010 (PMC13043496; doi:10.1016/j.jaacop.2025.11.010)
Supplement: Supplementary Table S1 [file mmc1.docx]

**SUPPLEMENTARY MATERIALS**

Table S1. Baseline and Weeks 6 and 12 BCDRS-R5 Internal Consistency and Scale Dimensionality and IRT

|  |  |  |  |  | **Factor 1 Pattern^a^** | | **IRT Parameter Estimate^b^** |
| --- | --- | --- | --- | --- | --- | --- | --- |
| **BCDRS-R_5_ Scale Items at**  **Baseline (N=296)** |  | **Mean** | ***SD*** | ***r*_it_** | **Loadings** | **Communalities** | **Slope** |
| Difficulty Having Fun |  | 2.113 | 1.280 | 0.569 | 0.790 | 0.624 | 1.408 |
| Social Withdrawal |  | 4.114 | 1.428 | 0.453 | 0.699 | 0.488 | 1.174 |
| Low Self-Esteem |  | 4.364 | 1.417 | 0.424 | 0.656 | 0.430 | 1.364 |
| Depressed Feelings |  | 4.905 | 1.078 | 0.438 | 0.654 | 0.428 | 1.547 |
| Depressed Facial Affect |  | 3.557 | 1.323 | 0.308 | 0.512 | 0.262 | 1.128 |
|  |  |  |  |  |  |  |  |
| Variance Explained by Factor | 44.68% |  |  |  |  |  |  |
| Cronbach's Coefficient α | 0.679 |  |  |  |  |  |  |
|  |  |  |  |  |  |  |  |
| **BCDRS-R_5_ Scale Items at**  **Week 6 (N=296)** |  | **Mean** | ***SD*** | ***r*_it_** | **Loadings** | **Communalities** | **Slope** |
| Difficulty Having Fun |  | 2.691 | 1.446 | 0.733 | 0.842 | 0.709 | 2.347 |
| Social Withdrawal |  | 2.528 | 1.366 | 0.716 | 0.832 | 0.692 | 2.445 |
| Low Self-Esteem |  | 2.821 | 1.528 | 0.605 | 0.736 | 0.542 | 1.755 |
| Depressed Feelings |  | 2.711 | 1.468 | 0.792 | 0.881 | 0.776 | 4.031 |
| Depressed Facial Affect |  | 2.280 | 1.053 | 0.605 | 0.742 | 0.550 | 1.955 |
|  |  |  |  |  |  |  |  |
| Variance Explained by Factor | 65.43% |  |  |  |  |  |  |
| Cronbach's Coefficient α | 0.863 |  |  |  |  |  |  |
|  |  |  |  |  |  |  |  |
| **BCDRS-R_5_ Scale Items at**  **Week 12 (N=296)** |  | **Mean** | ***SD*** | ***r*_it_** | **Loadings** | **Communalities** | **Slope** |
| Difficulty Having Fun |  | 2.198 | 1.372 | 0.749 | 0.849 | 0.721 | 2.670 |
| Social Withdrawal |  | 2.103 | 1.352 | 0.688 | 0.805 | 0.649 | 2.049 |
| Low Self-Esteem |  | 2.264 | 1.436 | 0.674 | 0.795 | 0.632 | 1.991 |
| Depressed Feelings |  | 2.094 | 1.320 | 0.754 | 0.857 | 0.736 | 3.569 |
| Depressed Facial Affect |  | 1.896 | 0.982 | 0.600 | 0.735 | 0.541 | 2.157 |
|  |  |  |  |  |  |  |  |
| Variance Explained by Factor | 65.61% |  |  |  |  |  |  |
| Cronbach's Coefficient α | 0.866 |  |  |  |  |  |  |

^a^ Extraction method was from a Principal Components Analysis (rotation not possible with 1 retained factor).

^b^ Item Response Theory (IRT) methods were implemented using the graded response model.
